# Immune Checkpoint Inhibitor-Related Sjögren’s Syndrome: An Ocular Immune-Related Adverse Event

**DOI:** 10.3390/diagnostics15091168

**Published:** 2025-05-04

**Authors:** Hideki Fukuoka, Akifumi Matsumoto, Chie Sotozono

**Affiliations:** Department of Ophthalmology, Kyoto Prefectural University of Medicine, Kyoto 602-8566, Japan; amatsu@koto.kpu-m.ac.jp (A.M.); csotozon@koto.kpu-m.ac.jp (C.S.)

**Keywords:** immune checkpoint inhibitor, pembrolizumab, Sjögren’s syndrome, keratoconjunctivitis sicca, immune-related adverse event (irAE), ocular toxicity, dry eye, fluorescein staining, Schirmer test, cancer immunotherapy

## Abstract

A 67-year-old male with metastatic human papillomavirus (HPV)-positive oropharyngeal cancer receiving pembrolizumab (anti-programmed cell death protein 1 [PD-1] immune checkpoint inhibitor) presented with bilateral ocular dryness. It is important to note that these symptoms appeared eight months after the initiation of the pembrolizumab therapy. Ophthalmologic evaluation revealed keratoconjunctivitis sicca with characteristic bulbar fluorescein staining and the Schirmer test showed 0 mm bilaterally. Serological testing demonstrated positive antinuclear and anti-SSb/La antibodies, consistent with Sjögren’s syndrome as an immune-related adverse event (irAE). Treatment with topical fluorometholone 0.1% and diquafosol 3% led to complete symptom resolution within one year while maintaining cancer immunotherapy. Long-term follow-up over 3.5 years demonstrated sustained ocular improvement and a favorable oncologic response without development of systemic autoimmune manifestations. This case highlights that Sjögren’s syndrome as an irAE may present with isolated ocular manifestations, which could be overlooked in clinical practice.

Immune checkpoint inhibitors have transformed cancer treatment by enhancing T-cell-mediated antitumor responses, demonstrating remarkable efficacy against various malignancies, including head and neck cancers. However, these therapeutic interventions have the potential to disrupt immune homeostasis, leading to immune-related adverse events (irAEs) that may affect multiple organ systems. Although ocular irAEs are less prevalent than other manifestations, they can have a substantial impact on patients’ quality of life and potentially result in permanent visual impairment if unrecognized. Sjögren’s-syndrome-like sicca syndrome has been documented as an irAE; however, isolated ocular presentations devoid of systemic manifestations remain under-researched in the literature.

The patient, diagnosed with HPV-positive oropharyngeal cancer two years prior, had no history of autoimmune disease. His cancer treatment history included paclitaxel, carboplatin, cetuximab, nivolumab, cisplatin, and etoposide before transitioning to pembrolizumab monotherapy (200 mg every three weeks) for metastatic disease. Notably, eight months after the initiation of this therapy, the patient exhibited symptoms of ocular dryness, devoid of the systemic manifestations characteristic of Sjögren’s syndrome.

A thorough ophthalmologic examination was conducted, revealing optimal visual acuity in both eyes. However, the examination also revealed minimal conjunctival hyperemia (black arrows) (Figure 1A), significant keratoconjunctivitis sicca, characterized by reduced tear meniscus (white arrowheads), and distinctive fluorescein staining patterns (white arrows) (Figure 1B). The complete absence of tear production following Schirmer testing (0 mm bilaterally) confirmed the severe aqueous deficiency. Laboratory evaluation revealed positive antinuclear antibodies, negative anti-SSa/Ro antibodies, and positive anti-SSb/La antibodies, thereby supporting the diagnosis of ocular immune checkpoint inhibitor-related Sjögren’s syndrome primarily manifesting with ocular symptoms.

Treatment with topical fluorometholone 0.1% was initiated while maintaining the pembrolizumab dosage. Despite the patient’s subjective improvement, the persistent keratoconjunctivitis sicca necessitated the addition of diquafosol ophthalmic solution 3%. Following one year of combined therapy, complete resolution of the symptoms and ocular manifestations was achieved (Figure 2A,B), enabling discontinuation of the eye drops while maintaining the cancer immunotherapy. The patient has been closely monitored with regular ophthalmologic examinations and has shown no recurrence of ocular symptoms over the follow-up period. Additionally, the patient has undergone computed tomography (CT) and positron emission tomography (PET) imaging evaluations every 12 months to assess the cancer response. After 3.5 years of continuous pembrolizumab therapy, a persistent reduction in the primary oropharyngeal tumor and mediastinal lymph node metastases has been maintained without the development of new metastatic lesions, suggesting a favorable long-term oncologic response.

Ocular irAEs are a growing indication of the complications associated with immune checkpoint inhibitor therapy. These events primarily affect the anterior segment, with dry eye syndrome and uveitis being the most common. Recent research suggests that ophthalmic irAEs may serve as predictors of clinical outcomes in patients undergoing immunotherapy [1]. A notable case report has documented bilateral keratitis and anterior uveitis induced by pembrolizumab, underscoring the potential for concurrent inflammatory conditions [2]. Several risk factors have been identified for the development of ocular irAEs, including prior ocular surgery, a history of trauma, and treatment with specific checkpoint inhibitor agents, such as pembrolizumab [3].

A number of clinical characteristics differentiate this case from primary Sjögren’s syndrome. The patient is male (although primary Sjögren’s syndrome predominantly affects females [4]), has no history of autoimmune disorders, and developed symptoms precisely eight months after the initiation of pembrolizumab. Furthermore, the isolated ocular presentation, devoid of xerostomia, exhibits greater consistency with the focal nature of early immune checkpoint inhibitor toxicity than with the typically more systemic manifestation of primary Sjögren’s syndrome. The patient’s keratoconjunctivitis sicca is strongly suspected to be a result of immune dysregulation induced by pembrolizumab, rather than a coincidental primary autoimmune condition. It is noteworthy that the onset of these symptoms occurred eight months after the commencement of pembrolizumab therapy, thereby establishing a clear temporal relationship that substantiates the diagnosis of an irAE.

Sjögren’s syndrome has been documented as an irAE associated with checkpoint inhibitors. However, manifestations that are limited solely to ocular symptoms are rare and may be overlooked in clinical practice [5,6,7]. This case underscores the necessity of monitoring for ocular irAEs in patients receiving immune checkpoint inhibitors, even in the absence of systemic manifestations.

In conclusion, this case exemplifies the effective management of pembrolizumab-induced Sjögren’s syndrome through the implementation of topical anti-inflammatory and secretagogue therapies, enabling the continuation of life-extending cancer immunotherapy. The successful long-term outcomes—in terms of both the sustained ocular symptom resolution and the favorable oncologic response—highlight that localized management of ocular irAEs can be effective without necessitating the discontinuation of immunotherapy. This approach may potentially contribute to sustained tumor control, as evidenced by the continued response observed in our patient after 3.5 years of treatment. It is recommended that patients receiving immune checkpoint inhibitors undergo regular ophthalmologic evaluations, even after initial symptom resolution, to monitor for late-onset manifestations or recurrence.

## Figures and Tables

**Figure 1:**
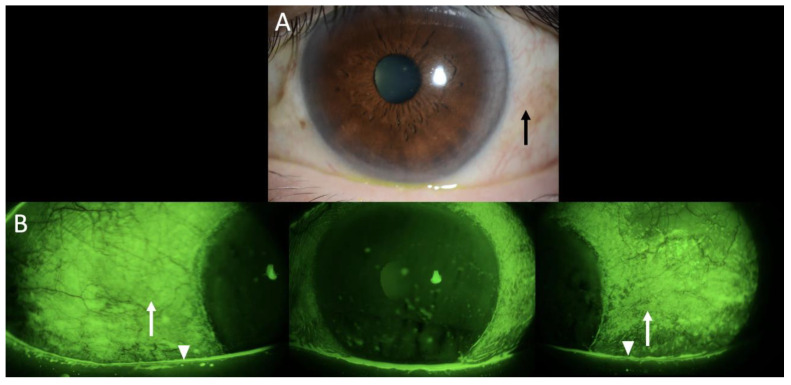
Slit photography and fluorescein staining in the left eye revealed minimal conjunctival hyperemia (black arrows), (**A**) superficial punctate epithelial erosions (white arrows), and a hallmark feature of keratoconjunctivitis sicca, accompanied by a reduced tear meniscus (white arrowheads) (**B**). Although similar findings were observed in the right eye, only the left eye is shown as a representative example. This finding was observed in a 67-year-old male who was undergoing treatment with pembrolizumab for human papillomavirus (HPV)-positive oropharyngeal cancer. It is noteworthy to observe the characteristic distribution of the staining within the bulbar conjunctiva.

**Figure 2:**
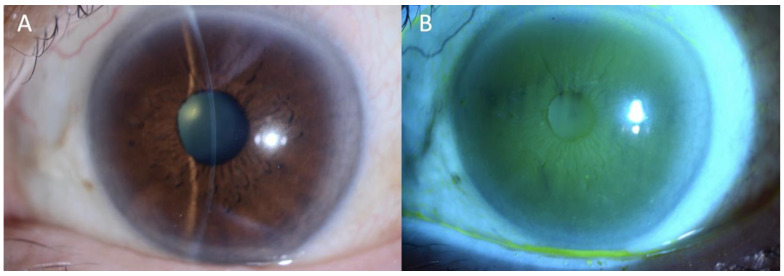
Slit photography and fluorescein staining in the left eye after one year of combined topical therapy. The images presented here illustrate the complete resolution of the pathological findings previously documented in Figure 1. A slit-lamp examination (**A**) revealed the restoration of normal conjunctival appearance without hyperemia, while fluorescein staining (**B**) demonstrated the absence of the characteristic punctate erosions and staining pattern that are hallmarks of Sjögren’s-syndrome-related keratoconjunctivitis sicca. This substantial enhancement was accomplished through the administration of fluorometholone 0.1% and diquafosol 3%, in conjunction with the ongoing treatment with pembrolizumab.

## Data Availability

The data presented in this study are available upon request from the corresponding author.
